# Adapted generative latent diffusion models for accurate pathological analysis in chest X-ray images

**DOI:** 10.1007/s11517-024-03056-5

**Published:** 2024-03-19

**Authors:** Daniel I. Morís, Joaquim de Moura, Jorge Novo, Marcos Ortega

**Affiliations:** 1 Centro de Investigación CITIC, Universidade da Coruña, A Coruña, Spain; 2 Grupo VARPA, Instituto de Investigación Biomédica de A Coruña (INIBIC), Universidade da Coruña, A Coruña, Spain

**Keywords:** Deep learning, Stable diffusion, Chest X-ray, Tuberculosis, Lung nodules

## Abstract

**Abstract:**

Respiratory diseases have a significant global impact, and assessing these conditions is crucial for improving patient outcomes. Chest X-ray is widely used for diagnosis, but expert evaluation can be challenging. Automatic computer-aided diagnosis methods can provide support for clinicians in these tasks. Deep learning has emerged as a set of algorithms with exceptional potential in such tasks. However, these algorithms require a vast amount of data, often scarce in medical imaging domains. In this work, a new data augmentation methodology based on adapted generative latent diffusion models is proposed to improve the performance of an automatic pathological screening in two high-impact scenarios: tuberculosis and lung nodules. The methodology is evaluated using three publicly available datasets, representative of real-world settings. An ablation study obtained the highest-performing image generation model configuration regarding the number of training steps. The results demonstrate that the novel set of generated images can improve the performance of the screening of these two highly relevant pathologies, obtaining an accuracy of 97.09%, 92.14% in each dataset of tuberculosis screening, respectively, and 82.19% in lung nodules. The proposal notably improves on previous image generation methods for data augmentation, highlighting the importance of the contribution in these critical public health challenges.

**Graphical abstract:**

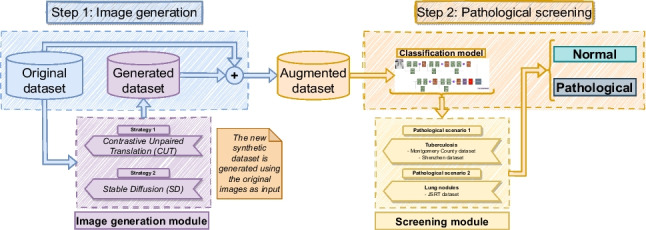

## Introduction

Respiratory diseases present a great global impact, causing about 1 million deaths annually in Europe [1]. Some of these diseases are acute, but there are also chronic conditions that can severely affect the life quality of their sufferers. Given the increase in life expectancy and the aging of the population, the mentioned diseases are even more prevalent and their impact will continue raising in the next decades. Lung tissue can be affected by many diseases and chronic conditions as asthma [2], cystic fibrosis [3], common flu [4], chronic obstructive pulmonary disease (COPD) [5], lung cancer [6], pneumonia [7], tuberculosis [8] or, more recently, COVID-19 [9]. Some of the most remarkable diseases among this group are lung cancer that represent a form of malignant lung nodules (the first cause of death by a respiratory condition with more than 2.2 million cases and more than 1.79 million deaths in 2020 [10]) and tuberculosis. In the case of lung nodules, it is worth to mention that many of them are benign but, given that they could be a sign of lung cancer, it is important to identify the actual underlying origin of the nodule and also to monitor its evolution [11]. Tuberculosis, which caused more than 1.6 million deaths worldwide in 2021 [12], stands out for its acute nature and ease of transmission, meaning that symptoms can improve or worsen very rapidly in short frames of time and lead to a rapid collapse of the healthcare services. In this context, clinicians require tools to quickly evaluate the state of each patient at a certain moment, to understand the extent of the pathology and to study its evolution.

The assessment of the pathological affectation on each patient can be performed using different techniques. In particular, the visualization of the affected area can be done with imaging modalities such as computerized tomography (CT) [13] or magnetic resonance imaging (MRI) [14]. However, despite these modalities present a great quality and level of detail, chest X-ray [15] is often used as the first imaging study given that the captures are cheaper and easier to perform. In a context of health emergency, CT or MRI are therefore an option to discard, as they would suppose a bottleneck to diagnose a reasonable amount of patients in a small amount of time. Instead, chest X-ray is a more appropriate image modality, given that it fits better to an emergency clinical scenario.

To support the tasks of the health workers, Computer-Aided Diagnosis (CAD) methods are extremely useful [16]. In the last years, these methods have been benefited from deep learning algorithms, which usually offer a better performance in comparison with classical methods [17]. However, the main issue that exists with this kind of strategy is the necessity of training with great amounts of labeled data, which is usually scarce in medical imaging domains. This is caused by the fact that manual labeling is a tedious, error-prone and time-consuming task that must be performed by professionals with a great experience in the field. To overcome this problem, many data augmentation strategies have been proposed in the state-of-the-art. The classical strategies of data augmentation include the modification of images with random trivial transformations such as rotations, translations or pixel intensity changes, among others [18]. However, the potential of these strategies is very limited, due to the lack of flexibility in their transformations. In particular, this limitation is caused by the lack of ability to learn image features by applying fixed transformations independently of the domain. In the case of medical imaging, this means that the data augmentation is unable to learn the specific characteristics of a given domain (for example, to understand those that are representative of a particular pathological scenario). Therefore, the differences that are present on the novel transformed images are usually irrelevant under a clinical point of view.

To solve the aforementioned issues, many efforts have been done in the last years to propose data augmentation alternatives, based on image generation [19]. The image generation models overcome the problem by learning the most relevant characteristics of a particular domain. In this way, the models are then able to randomly generate novel images that belong to the domain, reusing the previous knowledge. One of the most popular strategies is the use of Generative Adversarial Networks (GAN) [20]. A GAN is a deep network architecture composed of 2 different modules: the generator and the discriminator. The objective of the generator is to generate synthetic images with a realistic appearance, under the point of view of the discriminator. The most classical GAN approach consists on the generation of realistic images representative of a domain obtained from random noise. However, it has also been found that conditioning the generation model with an input image is useful to guide the generation process. In this scope, we can find the image translation models [21]. These proposals are able to convert input images from a certain domain to another different domain (for instance, in the field of medical imaging, it can convert the image of a normal patient to its hypothetical pathological version and vice versa). The potential of GAN architectures and image translation models was demonstrated in some medical imaging domains, such is the case of CT [22], brain MRI [23], or Optical Coherence Tomography (OCT) in the field of ophthalmology [24, 25].

However, despite the advantages of these data augmentation strategies, they still have some important points of improvement. In particular, these models show some issues like mode collapse (i.e., a situation where all the images generated by the model are very similar or identical) and training instability that is produced by the competition between the generator and the discriminator [26]. On their hand, the latent diffusion models present some improvements that can overcome those issues. Firstly, these models overcome the problem of mode collapse by including in their architectures several modules that allow better modeling of complex image distributions. Moreover, they can be trained without a discriminator, reducing the complexity of the loss functions that must be defined and tackling the problem of training instability, thanks to the removal of the competition between modules. These models also present other great advantages, like an inherent integration of Natural Language Processing (NLP) to the pipeline in the form of text prompts that helps to guide the training process, making the methodology more powerful. In the context of medical imaging, these text prompts can be obtained from the clinical history of the patients. This brings a great potential to the model, given that the imaging studies are usually accompanied by their corresponding clinical description in text, contributing with more useful information. In the last years, the research community has been benefited by new proposals of generative models with the aim to overcome the mentioned issues. In particular, diffusion models like Stable Diffusion [27] or DALL-E [28] have exceeded the performance of previous state-of-the-art approaches with GAN architectures in generic domains.

Nevertheless, despite all these advantages in generic domains, the quality of generated images can be rapidly identified as fake for many prompts, specially when those prompts are extremely complex and specific. This is understandable, given that the proposed latent diffusion models are originally conceived to accept any kind of prompt combination, while they can only be trained in a limited amount of prompts. Nonetheless, the generation process can be more manageable in a restricted domain, where only certain prompts will be inputted. This is the case of a specific biomedical imaging modality, which always represents the same reality, then having a more restricted data distribution, easier to derive, and a more manageable set of possible prompts, specially in a particular pathological scenario. Although recent progress in image generation approaches is both promising and intriguing, the current existing studies have yet to tackle the pressing challenges specific to tuberculosis and lung nodule screening. These highly relevant and prevalent pathologies demand tailored solutions, given their unique complexities and significant implications for public health. Furthermore, data scarcity is a crucial issue in these scenarios, as deep learning algorithms require large amounts of data to perform optimally, which is often unavailable in medical imaging domains.

In this work, we address a critical gap in the literature by proposing a novel chest X-ray data augmentation strategy based on image generation provided by latent diffusion models, in particular, the popular Stable Diffusion model. Our approach specifically targets two highly relevant and challenging pulmonary pathological scenarios: tuberculosis and lung nodules. By focusing on these scenarios, we aim to enhance the performance of automatic screening methods using chest X-ray images, ultimately benefiting patients, healthcare professionals, and public health systems. To achieve this, we fine-tune a generative latent diffusion model in both normal and pathological scenarios, generating useful synthetic chest X-ray images representative of the domain. We then integrate this novel set of synthetic images into the original dataset to bolster the performance of a fully automatic pathological screening method, implemented using a cutting-edge convolutional neural network architecture. We validate our proposal using three different representative public datasets affected by data scarcity, including two state-of-the-art public datasets for tuberculosis screening (Montgomery County and Shenzhen) and one additional dataset for lung nodule screening (JSRT). Our rigorous experimental design features an ablation study for each dataset, analyzing the data augmentation impact concerning the number of training steps for the latent diffusion model. Furthermore, we provide an incremental comparison of our methodology with the results of the baseline (i.e., using only classical data augmentation), CUT data augmentation, and latent diffusion data augmentation itself. The experimentation demonstrates the substantial potential of our proposed methodology, surpassing the performance of the baseline and classical image generation with CUT across all scenarios. This opens up the possibility of adding complementary information sources, like the image study reports, to continue improving that performance. This success indicates that our approach is suitable for extrapolation to other biomedical image modalities and domains, effectively addressing the gap in the literature while offering a valuable solution for data scarcity challenges in medical imaging.

The rest of the manuscript is structured as follows. In Section 2, we provide an insight of the works previously available in the state-of-the-art, closely related with the topic discussed in this manuscript. Then, in Section 3, we describe the 3 used public datasets, as well as the software and hardware resources that are necessary to reproduce the experimental procedure. After that, in Section 4, the proposed methodology is deeply explained, describing each of the followed steps: the first step of image generation in Section 4.1 and the second step of pathological screening in Section 4.2. In this section, we also explain the experimental details (Section 4.3) and the used evaluation metrics (Section 4.4). After that, the results of the experimental validation and their discussion are explained in Section 5. Finally, the main conclusions and possible lines of future work are explained in Section 6.

## Related works

The potential of image generation with GAN architectures has been proven in chest X-ray imaging. As reference, the work of Malygina et al. [29] proposes a method of image generation using an image translation model based on a GAN architecture, CycleGAN [30], to improve the performance of pneumonia classification in chest X-ray images. Particularly, in the mentioned work, CycleGAN is used to convert normal images to their hypothetical representation if they were pathological and vice versa. Moreover, Morís et al. [31] proposed a methodology of data augmentation using the same image translation architecture, CycleGAN, in portable chest X-ray images. In this case, the authors used a dataset with 3 different classes: normal, pathological and COVID-19. Therefore, they followed 6 different pathways of image translation: normal to pathological, normal to COVID-19, pathological to normal, pathological to COVID-19, COVID-19 to normal and COVID-19 to pathological. Later, the same authors demonstrated the adequacy of this data augmentation strategy in a real COVID-19 screening scenario [32]. In the case of Motamed et al. [33], the authors use chest X-ray images generated by a GAN architecture to improve the performance of COVID-19 and pneumonia screening. Another remarkable contribution in the state-of-the-art is the work of Morís et al. [34], which uses a Contrastive Unpaired Translation (CUT) [35] architecture for image-to-image translation in the context of tuberculosis. Particularly, authors train the CUT model with a reasonable amount of images from a tuberculosis dataset to convert in 2 pathways: normal to tuberculosis and tuberculosis to normal. Then, this pre-trained model is used to generate with 2 much smaller datasets. The final objective is to use this set of novel images to improve the performance of a tuberculosis screening method.

In the context of using latent diffusion models in medical imaging, some works have addressed this problem in biomedical domains like brain MRI [36]. Moreover, given the great availability of public chest X-ray datasets, some contributions can already be found in this field. In the case of Ali et al. [37], the authors propose a study of image generation using Stable Diffusion and DALL-E to obtain novel synthetic chest X-ray and CT images. Then, this set is shown to expert clinicians to identify those that have a realistic or fake appearance. The contribution of Packhäuser et al. [38] propose a method to generate anonymous synthetic chest X-ray images, excluding those synthetic images with patient-specific biometrics that have been reproduced from their corresponding real images, given that this could make it possible to identify the original patient (therefore, breaking the anonymity). The ultimate aim of this study is to evaluate the feasibility of using these generated images as exclusive training data for thoracic abnormality classification in a multipathological scenario, obtaining a competitive performance compared with a classifier trained with only real images. To this end, they compare 2 different approaches of image generation: a Generative Adversarial Network architecture and a Latent Diffusion Model. Furthermore, the approach of Chambon et al. [39] (named as *RoentGen*) refines a latent diffusion model in a large public chest X-ray imaging dataset with multiple pathologies using written medical reports as the input text prompt, aiming to generate realistic synthetic samples. The authors assess the quality of the generated images by qualitative visual inspection and also with quantitative metrics. Finally, the potential of *RoentGen* is also proven in a data augmentation scenario. Apart from RoentGen, Weber et al. [40] also explores the use of massive datasets to train latent diffusion models with chest X-ray images. In particular, the authors fuse other available public datasets to create the MaCheX dataset. Then, they propose a cascaded latent diffusion pipeline, called Cheff, that generates images in high-resolution leveraging a super-resolution module to refine the quality of the low-resolution scans. Lee et al. [41] propose UnixGen, a deep neural network based on a Transformer for the simultaneous generation of chest X-ray images and radiology reports.

## Materials

In this section, we present the materials used during the development of the work. In particular, Section 3.1 describes the 3 used datasets, while Section 3.2 defines the software and hardware considered for the methodological implementation.

### Datasets

**Fig. 1:**
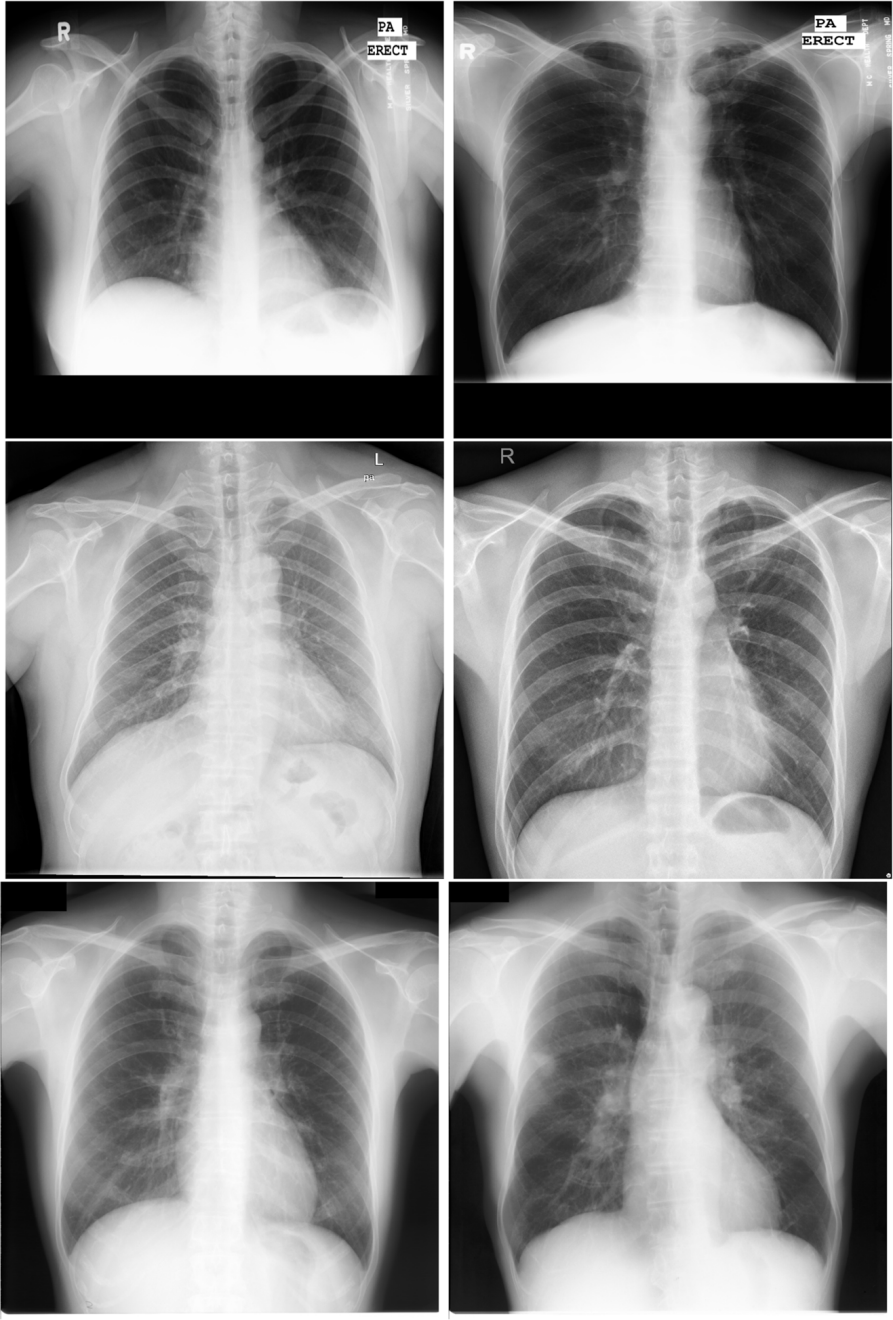
Representative examples of the used datasets of this work. The first column represents a normal case, while the second column represents a pathological case (tuberculosis or lung nodules, depending on the dataset). First row: examples of the Montgomery dataset (tuberculosis). Second row: examples of the Shenzhen dataset (tuberculosis). Third row: examples of the JSRT dataset (lung nodules)

For the aims of this work, 3 different public datasets have been used, representative of 2 different pathological scenarios of reference. Firstly, in the case of the tuberculosis screening, the Montgomery County (MC) dataset [42] and the Shenzhen dataset [42] were chosen given that they are representative of a small-sized and a medium-sized dataset, respectively. Secondly, in the case of lung nodule screening, the JSRT dataset was chosen [43]. The reason to use these datasets is that they are representative of each pathological scenario and suffer from data scarcity. This is a suitable scenario to evaluate the capabilities of the image generation models to perform data augmentation. Some examples of these datasets can be seen in Fig. 1. The description of each dataset is detailed below:

- **Montgomery County (MC) dataset (available at** [44]**):** this public dataset was retrieved by the Department of Health and Human Services of Montgomery County (Maryland, USA) and is composed of 80 normal cases and 58 pathological cases (i.e., with evidences of tuberculosis affectation), making a total of 138 images. These images correspond with posterior-anterior captures with variable resolutions of 4020 $\times$ 4892 or 4892 $\times$ 4020 pixels.
- **Shenzhen dataset (available at** [45]**):** this dataset was obtained by the staff of the Shenzhen Hospital (China), as part of routine care. It is composed of 326 normal cases and 336 cases with evidences of tuberculosis affectation, making a total of 662 captures. The images present variable resolutions that range from 948 $\times$ 1130 to 3001 $\times$ 3001 pixels.
- **JSRT dataset (available at** [46]**):** this dataset is composed of conventional chest X-ray captures that were digitized with a resolution of 2048 $\times$ 2048 pixels. In total, it includes 247 images, having 154 cases with lung nodules and 93 without lung nodules. Particularly, from the 154 pathological cases, 100 are malignant and 54 are benign.

### Software and hardware resources

In this section, we detail the software and hardware resources considered for the implementation of the methodology. The used software libraries can be seen in Table 1. In this regard, the implementation of the first step of the methodology (image generation) has been mainly based on several Hugging Face libraries that support the process of Stable Diffusion models deployment, fine-tuning and inference. In the case of the screening, the implementation was mainly supported by the library Tensorflow. Furthermore, the details of the used hardware can be seen in Table 2. To speed up the processes of training and inference, the experimentation was performed using an NVIDIA Tesla A100 with 2 GPUs of 80 GB each, although only one at a time was needed to carry out the experiments. Moreover, the used driver version is the 460.160.00.

**Table 1:** Required software libraries necessary for the reproducibility of the methodology presented in this work

Name	Version	Description
Accelerate	0.16.0	This library enables to work with PyTorch training loops easily
Diffusers	0.13.1	Provides pre-trained vision and audio diffusion models
Matplotlib	3.6.1	This library enables the graphical visualization of data
Numpy	1.23.4	Widely used Python library to work with arrays
Pillow	9.2.0	Pillow provides useful functionalities to work with images in Python
Torch	1.12.1+cu116	Popular library to work with deep learning algorithms
Torchvision	0.13.1+cu116	Torchvision adds useful functionalities to torch
Transformers	4.26.1	Allows to work with state-of-the-art transformer architectures easily
Scikit-image	0.19.3	This library provides a set of useful function to work with images
Scikit-learn	1.1.2	Scikit-learn is a library to implement machine learning methods
Tensorflow	2.11.0	This library represents a deep learning framework

**Table 2:** Hardware required for the development of this methodology

Name	Description
OS	Ubuntu 20.04.5 LTS (Focal Fossa)
Kernel	5.4.0-131-generic
Architecture	x86-64
CPU	AMD EPYC 7763 64-Core Processor
RAM	503.9 GiB
Hard disk	1007 GB

## Methodology

**Fig. 2:**
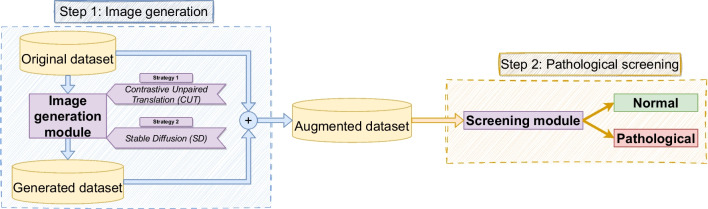
Detail of the methodology proposed in this work with 2 different steps (image generation and Pathological screening)

In this section, we present our proposed methodology, which consists of two distinct steps illustrated in Fig. 2. In the first step, the models for image generation were separately trained for each dataset and pathological scenario, following 2 different strategies: Contrastive Unpaired Translation (CUT) and Stable Diffusion (SD) Then, these trained models are used to generate the novel sets of synthetic images. Finally, in the second step, the generated set is added to the original dataset. This augmented version of the original dataset is fed into the screening model to distinguish between normal and the corresponding pathological scenario (tuberculosis or lung nodules). To ensure a fair comparison with previous approaches, we clarify that the screening model is trained using the augmented version of the dataset, while only the original images are employed in the test set. This distinction is crucial to maintain the integrity of our experimental design and provide an accurate evaluation of our methodology’s performance in comparison to the existing methods.

### 1^st^ step: image generation

In this part of the methodology, we present the 2 image generation strategies that were followed. Firstly, the CUT is presented, representing a classical approach of image generation that is based on a Generative Adversarial Network framework. Secondly, the Stable Diffusion model is deeply explained, which represents the latent diffusion approach for image generation.

**Fig. 3:**
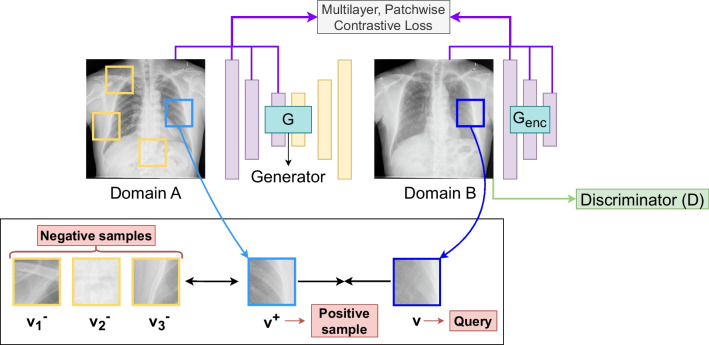
Structure of the contrastive unpaired translation model, showing the patches that are compared to compute the loss

**Strategy 1: Contrastive Unpaired Translation (CUT).** This architecture, which is depicted in Fig. 3, has an encoder-decoder generator *G* based on a ResNet of 6 blocks and a discriminator *D*, based on a $70 \times 70$ PatchGAN. *G* receives an image *a* from domain *A* as input and returns an image $\hat{b}$ which is the transformation of the input that presumably should belong to class *B*. To train the CUT model, it is necessary to define the loss function, which is expressed as the combination of different components. The first component is the adversarial loss $\mathcal {L}_{GAN}$, which is computed from the discriminator output using the expression of Mean Squared Error (MSE) loss, whose definition can be seen in Eq. 1. The objective of the discriminator is to classify the generated images as fake (label 1) and the original images as real (label 0).

1 $$\begin{aligned} \mathcal {L}_{GAN} (G, D, A, B) \!=\! \mathbb {E}_{b \sim B} (D(b))^2 + \mathbb {E}_{a \sim A} (D(G(a)) - 1)^{2} \end{aligned}$$

Secondly, it is necessary to define PatchNCE loss (denoted as $\mathcal {L}_{PatchNCE}$) the loss component that is based on the idea of contrastive learning. In this kind of learning, given the original image and its translation, the idea is to maximize the association between 1 patch of the translated image (known as query) and the corresponding patch of the original image (known as positive sample). At the same time, the model must also minimize the association between the query and a number *N* of negative samples (i.e., other random patches different from the positive sample). The comparison between patches is performed using the weights of some layers from the generator encoder, which are used as representative features of each patch. The layers chosen from the encoder are then connected to *H*, a multilayer perceptron (MLP) of 2 layers, resulting in a K-dimensional vector that represents each patch. In this way, the comparison between patches is performed calculating the cosine similarity between the K-dimensional vector of the query and the positive sample as well as between the vectors of the query and the *N* negative samples. This difference is scaled with a temperature value of $\tau$ = 0.07, in the same line as proposed in Park et al. [35]. Then, these values are concatenated and a softmax function is applied. Denoting the query as *v*, its corresponding positive sample as $v^{+}$ and the set of negative samples as $v^{-}$, the expression of the contrastive loss can be defined as seen in Eq. 2.

2 $$\begin{aligned} l(v, v^{+}, v^{-}) = -\log \left[ \frac{\exp (v \cdot v^{+})/\tau }{\exp (v \cdot v^{+}/\tau ) + \sum _{n = 1}^{N} \exp (v \cdot v_{n}^{-}/\tau )}\right] \end{aligned}$$

As a result from this step, we obtain a stack of features $\{z_{l}\}_{L} = \{H_{l}(G_{l}^{enc}(a))\}_{L}$. In this expression, *l* denotes a specific layer (where each layer has a particular number of spatial locations $S_{l}$) and *L* the whole amount of layers. In this way, the PatchNCE loss can be defined as shown in Eq. 3.

3 $$\begin{aligned} \mathcal {L}_{PatchNCE} (G, H, A) = \mathbb {E}_{a \sim A} \sum _{l = 1}^{L} \sum _{s = 1}^{S_{l}} l(\hat{z}_{l}^{s}, z_{l}^{s}, z_{l}^{S\backslash {}s}) \end{aligned}$$

**Fig. 4:**
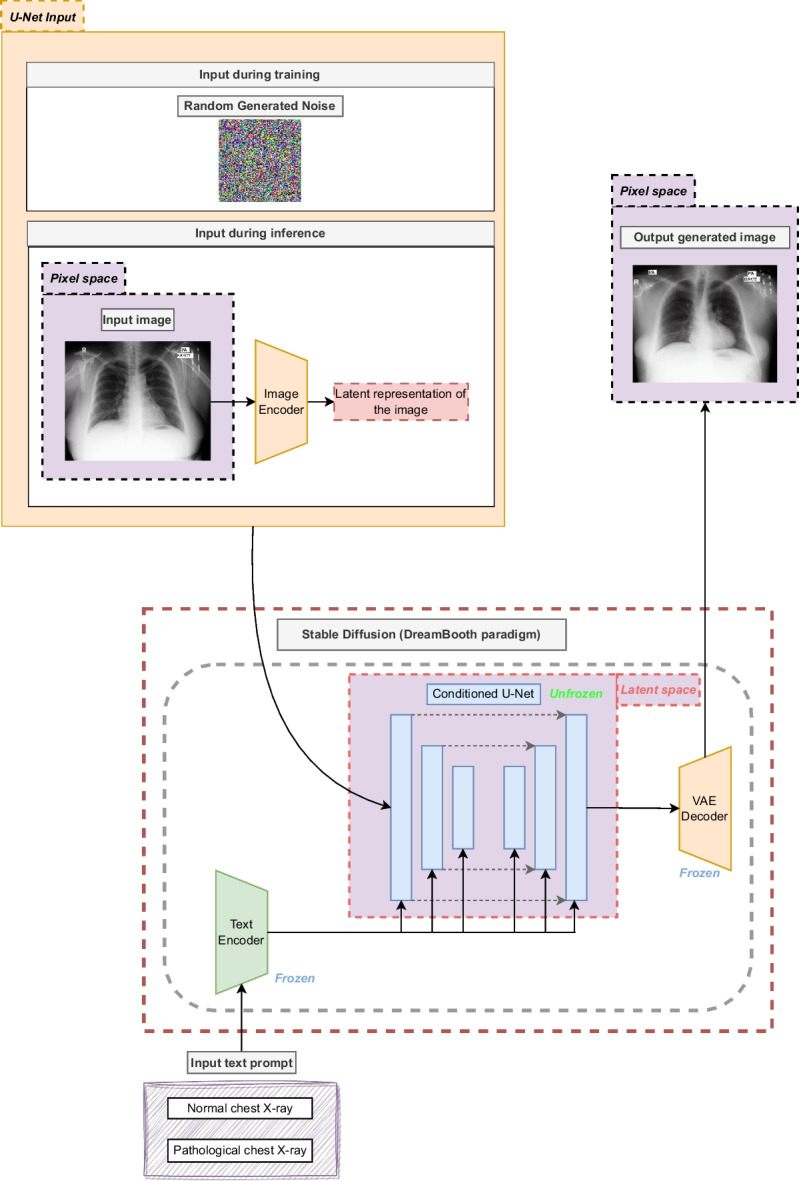
Architecture of the adapted Stable Diffusion model following the DreamBooth training framework (keeping the U-Net unfrozen but the text encoder and the VAE frozen). It must be noted that the U-Net input vector is different in training and testing

The adversarial loss and the PatchNCE loss are then combined to form a joint expression. Other important element that is added to the CUT training is the identity loss, which is computed as the PatchNCE loss of the images in the class B (denoted as $\mathcal {L}_{PatchNCE}$(G, H, B)). The inclusion of an identity loss prevents the model to generate unexpected changes on images. The contribution of $\mathcal {L}_{PatchNCE}$(G, H, A) and $\mathcal {L}_{PatchNCE}$(G, H, B) can be weighted with $\lambda _{A}$ and $\lambda _{B}$, respectively. In this work, it was decided to give an equal balance to both components. Therefore, both $\lambda _{A}$ and $\lambda _{B}$ will be set to 1.0. Once all these expressions are defined, they are joined as can be seen in Eq. 4. It must be mentioned that this is the loss function used to train the generator while the discriminator is only trained with the adversarial loss.

4 $$\begin{aligned} \mathcal {L} =&\mathcal {L}_{GAN}(G, D, A, B) + \lambda _{A} \mathcal {L}_{PatchNCE}(G, H, A)\nonumber \\&+ \lambda _{B} \mathcal {L}_{PatchNCE}(G, H, B) \end{aligned}$$

**Strategy 2: stable diffusion** The adaption of this second strategy is proposed as an alternative from the classical image generation architectures based on GANs. For that reason, we consider the use of a popular latent diffusion model known as Stable Diffusion [47]. In particular, in this work, the chosen version is the 1.4, given that it was the most established version at the moment that the experimentation was performed. This architecture of image generation consists of 3 different components that can be seen in Fig. 4: a Variational Autoencoder (VAE) [48], a conditioned U-Net [49] and a text encoder CLIP ViT-L/14 [50] as the conditioning mechanism. There are several ways to fine-tune the model, depending on the components that are kept frozen (i.e., that are kept as in their pre-trained state, without refinement) and unfrozen. Particularly, in this work, we considered the framework DreamBooth [51], which freezes the VAE and the text encoder, putting the focus on the U-Net model. The training process of the Stable Diffusion model represents a great advantage in the context of image generation models in comparison with similar approaches like DALL-E, which work on pixel space with the whole image as input, making the algorithm very expensive in terms of memory and time requirements. To avoid this problem, the Stable Diffusion model compresses the dimensionality of the input image into a set of embeddings in the latent space. This considerably reduces the amount of required memory, speeding up the training process as well. The contribution of each module of the architecture is explained below.

- Text encoder (CLIP ViT-L/14): This module converts the input text to a latent representation, making possible to implement a conditioning mechanism for the U-Net architecture and guide the training process to obtain the expected generated image.
- Conditioned U-Net: The target of the U-Net module is to iteratively denoise an initial vector that belongs to the latent space. In the original concept of Stable Diffusion, whose aim is to perform text-to-image generation, the input of this module is a vector randomly generated from a gaussian distribution. It is important to mention that this denoising process occurs entirely in the latent space. With regard to the conditioning part of this module, it is necessary to add the information of the text prompt to influence the input of the U-Net. This information is provided by the latent representation of the text prompt provided by the text encoder.
- Variational Autoencoder (VAE): The encoder of the VAE has the ability to compress an image from its high-dimensional representation to a latent representation of lower dimensionality. Then, the decoder of this component is able to recover the high-dimensionality from the latent representation of the image. The main difference between a VAE and a conventional autoencoder is that the VAE represents the latent space as a Gaussian distribution of the data instead of being represented as a single point. This is extremely helpful in the context of image generation, because it enables the model to retrieve a new sample close to the distribution of the original data.

To train the Stable Diffusion model, it is necessary to follow several steps. Firstly, both the images from the original dataset that must be learned, and the input text prompt must be encoded. Nevertheless, it is also necessary to feed the generation model with noise. This noise will be randomly generated from a gaussian distribution in the latent space with standard parameters of $\mu = 0$ and $\sigma = 1$. Denoting *N* as the random noise, $\mathcal {N}$ as an arbitrary normal distribution, 0 as a zero matrix, *I* as the identity matrix and (*h*, *w*) as the dimensions of the latent space, the random noise latent vector can be represented as in Eq. 5:

5 $$\begin{aligned} N \sim \mathcal {N}(0_{(h, w)}, I_{(h, w)^2}) \end{aligned}$$

Once this expression is defined, the input of the conditioned U-Net can be defined as follows. As mentioned before, the text prompt (denoted as $x_{input}$) and the image of the input dataset (denoted as $y_{target}$) must be encoded. In the case of the text prompt, this is performed with the text encoder (a process that is defined as $Enc_{text}(x_{input})$) while, in the case of the image, it is performed with the VAE encoder (a process that is defined as $VAE(y_{target})$). Furthermore, on each diffusion step *t*, a new random noise vector N will be obtained and combined with the image encoding, using the operator $\oplus _{t}$. With all of these points defined, the noise $\hat{N}$ predicted by the U-Net (denoted as Unet in this occasion) on each step *t* is defined as in Eq. 6:

6 $$\begin{aligned} \hat{N} = Unet(Enc_{text}(x_{input}), VAE (y_{target}) \oplus _{t} N, t) \end{aligned}$$

To optimize the weights of the modules, it is necessary to define the loss function. The main objective of Stable Diffusion is to predict the noise found on an image. Therefore, the loss function will be calculated as the difference between the output of the U-Net and the actual noise of the step *t*. In the same line as in the original work, we decided to use the MSE function as the loss, which computes the difference between *N* and $\hat{N}$. The full loss expression of this objective, denoted as $\mathcal {L}$, can be seen in Eq. 7.

7 $$\begin{aligned} \mathcal {L} = \frac{1}{h \times w} \sum _{i=0}^{h} \sum _{j=0}^{w} (\hat{N}_{i, j} - N_{i, j})^2 \end{aligned}$$

It is important to note that the methodology herein proposed makes a slight change over the original Stable Diffusion text-to-image paradigm with regard to inference. In particular, it is desirable to guide the process not only with text prompts, but also with real samples of the original dataset (a pipeline that could be defined as image generation conditioned by image and text). Therefore, random generated gaussian noise must be replaced with the latent representation of the input image obtained by an image encoder.

**Fig. 5:**
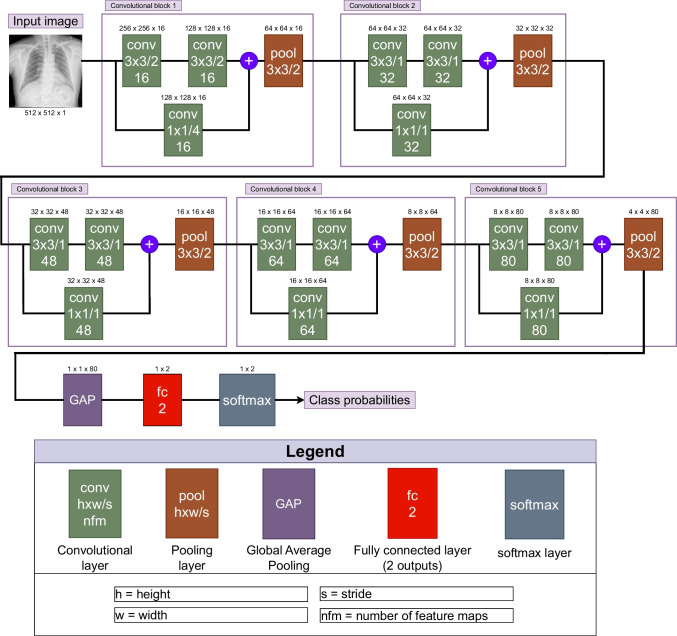
Detailed description of the classification deep network architecture used for the screening task. The intermediate output sizes are shown at the top of each layer

### 2^nd^ step: Pathological screening

The deep network architecture to perform the screening task is depicted in Fig. 5. In particular, we adapt the same architecture used in Pasa et al. [52] for tuberculosis screening. Globally, this architecture is structured in 5 general convolutional blocks, which are composed of several convolutional layers, ending with a pooling layer. These pooling layers are necessary to reduce the dimensionality, although this effect is also achieved for the first convolutional block using a stride bigger than one in the convolutional layers. In particular, the convolutional blocks have been designed to reduce the dimensionality by 2, except in the first block, where the magnitude of reduction is 8 times. Furthermore, each convolutional block has 2 different parallel pathways, one that applies 2 sequential convolutional layers with kernel size of 3 $\times$ 3 and an additional residual connection that applies a single convolution with a kernel size of 1 $\times$ 1, where all the convolutional layers make use of batch normalization and are zero-padded to avoid losing additional dimensionality. Before the pooling layer is applied, the outputs obtained from both pathways are summed. The number of feature maps on each convolution block is an incremental multiple of 16, having 16 for the first block, 32 for the second, 48 for the third, 64 for the fourth and 80 for the fifth one. Immediately after all the convolutional blocks, the architecture applies a global average pooling (denoted as GAP), followed by a fully convolutional layer of 2 outputs. Finally, the model applies a softmax function to obtain the class probabilities.

### Experimental details

With regard to the training of the image generation models, it is important to clarify some aspects. Firstly, the CUT model is only trained for the JSRT dataset, given that there were no previous reference values with this data augmentation approach. Moreover, another detail that must be remarked is that, in that previous work of the state-of-the-art, the CUT model was trained on a large-scale chest X-ray dataset of tuberculosis, the TBX11K [53]. In the particular case of the JSRT dataset, the training and inference of the CUT model was made with that dataset itself. Following the same training details of the previous state-of-the-art approach, the CUT model was trained during 200 epochs with the Adam algorithm [54], a mini-batch size of 1 and a constant learning rate of $\alpha = 0.0002$.

On the other hand, in the case of Stable Diffusion, the parameters for DreamBooth are set to a constant learning rate of $5 \times 10^{-6}$, a mini-batch size of 1 and a variable number of training steps that will depend on each experiment. In particular, 2 different models were trained: a model that represents the normal class and another model that represents the pathological class (*i.e.,* tuberculosis or lung nodules, depending on the used dataset). In this case, the considered text prompts were the class of the samples. Therefore, for normal cases, the model was trained with the prompt < *normal chest X-ray* > while for pathological cases the model was trained with the prompt < *patho chest X-ray* >.

In the case of the screening model, it is important to note that, to perform a fair comparison with the baseline method (i.e., without the proposed data augmentation), the model is trained with both original and synthetic images, but the validation set will only be composed of original images. Regarding the training details of the screening model, the validation process was performed with a random 5-fold cross validation. For each fold, the model is trained during 500 epochs, optimizing the weights with the Adam algorithm. In particular, the learning rate was set to a constant value of $8 \times 10^{-5}$, $\beta _{1}$ was set to 0.9, $\beta _{2}$ to 0.999 and $\epsilon$ to $1 \times 10^{-8}$. To have a global summary of the model performance, the mean and the standard deviation among the 5 folds is calculated for each metric.

### Evaluation metrics

To evaluate the screening models, we considered some of the most typical metrics used in the state-of-the-art for classification problems: accuracy (abbreviated as ACC), recall (abbreviated as RECA), specificity (abbreviated as SPEC), precision (abbreviated as PREC), F1-Score (abbreviated as F1-SC) and AUC-ROC (abbreviated as AUC). Defining TP as True Positives, TN as True Negatives, FP as False Positives and FN as False Negatives, the expression of the mentioned evaluation metrics can be seen in Eqs. 8, 9, 10, 11, and 12, respectively:

8 $$\begin{aligned} ACC = \frac{TP + TN}{TP + FP + TN + FN} \end{aligned}$$

9 $$\begin{aligned} RECA = \frac{TP}{TP + FN} \end{aligned}$$

10 $$\begin{aligned} SPEC = \frac{TN}{TN + FP} \end{aligned}$$

11 $$\begin{aligned} PREC = \frac{TP}{TP + FP} \end{aligned}$$

12 $$\begin{aligned} F1-SC = \frac{2 * PREC * RECA}{PREC + RECA} \end{aligned}$$

In the case of AUC-ROC, this metric is calculated as the area under the ROC curve [55]. In particular, ROC curve is an exhaustive metric given that it evaluates the global performance of the model considering different operation points. The expression of this metric can be seen in Eq. 13.

13 $$\begin{aligned} AUC = \int _{0}^{1} TPR \cdot d(FPR) \end{aligned}$$

## Results and discussion

In this section, we present the results obtained from our proposed methodology and discuss their implications. Our experimentation focuses on the impact of incorporating novel sets of synthetic images as a data augmentation strategy to enhance the automatic screening of two highly relevant pathological scenarios: tuberculosis and lung nodules. To achieve this, we utilize three public representative datasets facing data scarcity issues: Montgomery County and Shenzhen for tuberculosis screening, and JSRT dataset for lung nodule screening. In all cases, the training process incorporates both the original and synthetic images, while the testing is conducted exclusively with the original images. This approach ensures a fair comparison with previous data augmentation methods and allows us to accurately evaluate the performance and efficacy of our methodology.

Particularly, we performed an exhaustive experimentation using 3 public representative datasets. This experimentation includes an ablation study for all the scenarios, to choose the most appropriate configuration of the Stable Diffusion model regarding the number of training steps and an additional study that analyzes the quality of the generated images under a qualitative point of view. The ablation studies are necessary because it is well-known that selecting the appropriate amount of training steps for an image generation model is challenging and no straightforward. For this reason, we have selected several fixed numbers of training steps, to study the performance evolution. This performance will be evaluated as an incremental approach, comparing the performance of the Stable Diffusion augmentation with a baseline (with only classical data augmentation) and the CUT augmentation. Regarding the ablation study, we chose 4 configurations with Stable Diffusion, after training during 2500, 5000, 7500 and 10,000 steps. Furthermore, in the particular case of the JSRT dataset, it was necessary to include an additional configuration, training during 12,500 steps, to ensure a convergence of the global performance.

This section is structured as follows. Firstly, Section 5.1 shows the results obtained for the Montgomery dataset and their discussion, Section 5.2 for the Shenzhen dataset and Section 5.3 for the JSRT dataset. Moreover, in Section 5.4, we also provide a qualitative analysis of the generated images that were obtained and, finally, in Section 5.5 we discuss the results of the proposed methodology with similar works found in the state-of-the-art.

### 1^st^ experiment: Montgomery dataset (automatic tuberculosis screening)

**Fig. 6:**
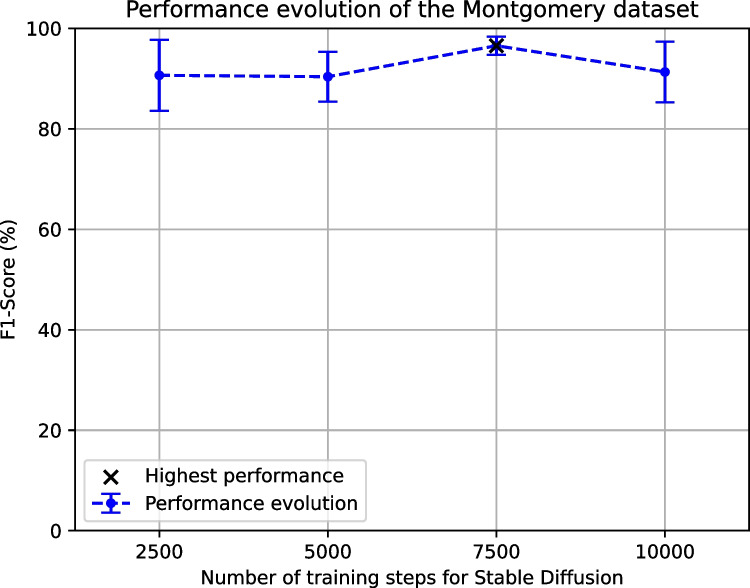
Evolution of the F1-Score for the tuberculosis screening using the Montgomery dataset when adding the images generated by the Stable Diffusion model to the baseline, when trained during 2500, 5000, 7500 and 10,000 steps, respectively

The evolution of the results that were obtained for the tuberculosis screening using the Montgomery dataset can be seen in Fig. 6. This evolution shows a very similar performance regarding the mean value of F1-Score between the configuration with 2500 and 5000 training steps, but with an improvement in terms of the standard deviation. For 7500 steps, the improvement is notable, regarding the mean F1-Score and its standard deviation. However, after this amount of steps, the performance converges and the F1-Score notably drops with 10,000 training steps. This leaves 7500 training steps as the highest-performing configuration.

Moreover, Table 3 shows the comparison between the results obtained in the work of Morís et al. [34] (for both the baseline and the CUT) with the highest-performing Stable Diffusion configuration (with 7500 training steps, as previously stated). This comparison obtains a remarkable improvement of the performance regarding all the metrics. Globally, the mean value of accuracy raises from 88.35% (Baseline) and 88.41% (CUT) to a 97.09%. This is also seen in terms of F1-Score, with an improvement from 82.89% (Baseline) and 86.32% (CUT) to 96.54%. A similar conclusion can be extracted from AUC-ROC, which goes from 0.8652 (Baseline) and 0.8713 (CUT) to 0.9703. Regarding the individual metrics recall, precision and specificity, the performance improvement is also noticeable. In particular, recall and precision go from less than 90% to 96.57% and 96.80%, respectively. In the case of the specificity, which was lower for the CUT (89.38%) in comparison with the baseline (91.53%), there is a raise to 97.49% for the Stable Diffusion model.

**Table 3:** Comparison of the results obtained for the automatic tuberculosis screening using the Montgomery dataset with the different chosen approaches: the baseline and the approach with CUT data augmentation against the highest-performing approach of data augmentation with Stable Diffusion (our proposal)

Method	ACC	RECA	SPEC	PREC	F1-SC	AUC
Baseline [34]	88.35% ± 6.28%	81.80% ± 12.04%	91.53% ± 6.95%	86.35% ± 13.77%	82.89% ± 9.64%	0.8652 ± 0.0784
CUT [34]	88.41% ± 5.27%	88.59% ± 9.88%	89.38% ± 9.07%	86.24% ± 12.04%	86.32% ± 6.37%	0.8713 ± 0.0707
Ours	**97.09%** ± **1.46%**	**96.57%** ± **4.29%**	**97.49%** ± **3.08%**	**96.80%** ± **3.93%**	**96.54%** ± **1.81%**	**0**.**9703** ± **0**.**0164**

The highest results for each metric (i.e., those with the highest mean value) are highlighted in bold

The improvements in terms of standard deviation are also quite notable. By the means of global metrics, the standard deviation of the accuracy lowers from 6.28% (baseline) and 5.27% (CUT) to a 1.46%, the standard deviation of F1-Score from 9.64% and 6.37% to 1.81% and the same value for AUC goes from 0.0784 (baseline) and 0.0707 (CUT) to 0.0164. Recall and specificity also demonstrate a great improvement in robustness. In the case of the recall, there is an improvement from 12.04% (baseline) and 9.88% (CUT) to 4.29%. Regarding precision, the standard deviation goes from 13.77% (baseline) and 12.04% (CUT) to 3.93%. Finally, regarding specificity, the standard deviation that was even higher with the CUT (going from 6.95% to 9.07% in comparison with the baseline) improves in the case of Stable Diffusion, achieving a 3.08%. From these results, several conclusions can be obtained. Firstly, it is demonstrated that the Stable Diffusion models generate useful images that can improve the performance of the tuberculosis screening in this particular scenario. Secondly, the results obtained by Stable Diffusion prove to be higher than those obtained by the Contrastive Unpaired Translation. Finally, it is worth to mention the high robustness demonstrated by the Stable Diffusion augmentation, notably improving the standard deviation for all metrics.

### 2^nd^ experiment: Shenzhen dataset (automatic tuberculosis screening)

**Fig. 7:**
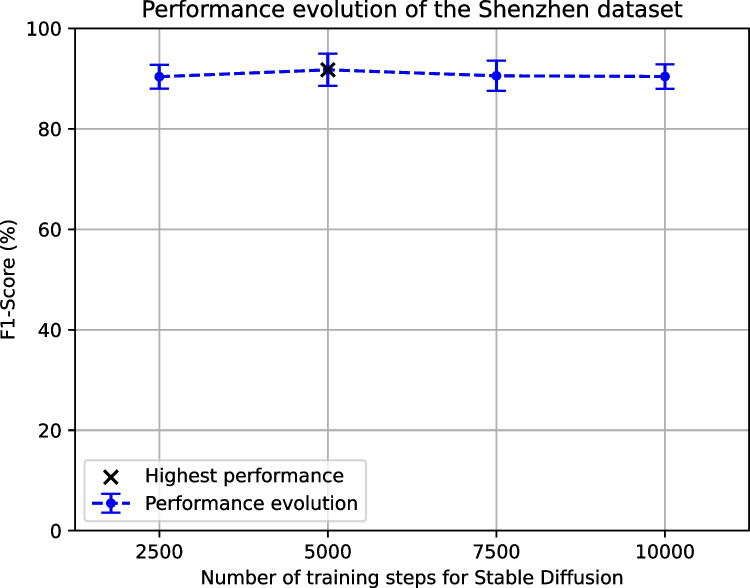
Evolution of the automatic tuberculosis screening performance in terms of the F1-Score when adding the generated images to the Shenzhen dataset with the different chosen configurations of Stable Diffusion (with 2500, 5000, 7500 and 10,000 training steps)

In the case of the automatic tuberculosis screening using the Shenzhen dataset, the evolution of the F1-Score after the generated images are added can be seen in Fig. 7. This evolution depicts an improvement of the F1-Score between 2500 and 5000 training steps. Then, this performance rapidly starts to drop for 7500 and 10,000 steps. Moreover, the comparison between this approach, using the highest-performing Stable Diffusion configuration (5000 training steps), and the proposals of Morís et al. [34] (the baseline and the CUT) can be seen in Table 4. Those results depict an improvement of all the metrics. In particular, the mean accuracy goes from 89.42% (baseline) and 90.33% (CUT) to 92.14%. In the case of the recall, the improvement that was slight in the case of the CUT (going from 88.11% to 88.40%) raises to 89.23% for our approach. The specificity and precision have a very similar improvement, as they go from about 90% for the baseline and a 92% for the CUT to a 94.71% and a 94.57%, respectively. Regarding the value of AUC, this improvement goes from 0.8846 in the baseline and 0.9088 with the CUT to a 0.9197 in the case of Stable Diffusion. In the same line as in the previous experiment, it can be concluded that the Stable Diffusion is able to generate relevant images to improve the performance of an automatic tuberculosis screening model. In the same way, the data augmentation provided by Stable Diffusion proves to be more powerful than the one provided by the CUT model.

**Table 4:** Comparison of the results obtained with the different approaches for the automatic tuberculosis screening using the Shenzhen dataset: the baseline, the data augmentation with CUT and the data augmentation using the Stable Diffusion (our proposal) model with the highest-performing configuration

Method	ACC	RECA	SPEC	PREC	F1-SC	AUC
Baseline [34]	89.42% ± 2.30%	88.11% ± 3.42%	90.60% ± 4.44%	90.90% ± 3.23%	89.41% ± 2.16%	0.8846 ± 0.0379
CUT [34]	90.33 ± 1.41%	88.40% ± 2.51%	92.21% ± 2.18%	92.28% ± 1.22%	90.27% ± 1.17%	0.9088 ± 0.0229
Ours	**92.14%** ± **2.22%**	**89.23%** ± **5.66%**	**94.71%** ± **0.92%**	**94.57%** ± **0.90%**	**91.74%** ± **3.21%**	**0**.**9197** ± **0**.**0254**

The highest results for each metric (i.e., those with the highest mean value) are highlighted in bold

### 3^rd^ experiment: JSRT dataset (automatic lung nodule screening)

**Fig. 8:**
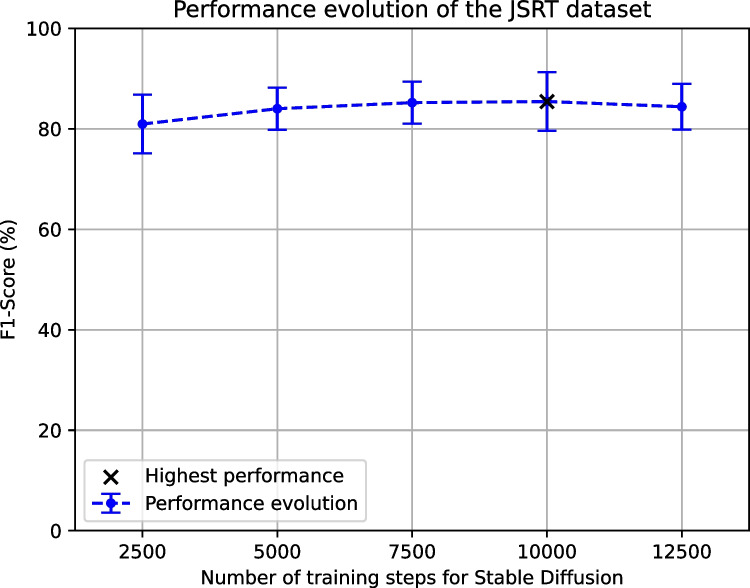
Evolution of the F1-Score for the lung nodule screening (JSRT dataset) when adding the novel set of generated images regarding the different configurations of the Stable Diffusion model (with 2500, 5000, 7500, 10,000 and 12,500 training steps)

Regarding the screening of lung nodules using the JSRT dataset, the evolution of the F1-Score when adding the images generated by the Stable Diffusion with the different configurations can be seen in Fig. 8. The evolution shows a raise of the F1-Score until 10,000 training steps that, nevertheless, starts to drop once reached the number of 12,500 training steps, leaving the configuration of 10,000 training steps as the highest-performing scenario. The comparison of the results obtained by this highest-performing Stable Diffusion configuration with the Baseline and the CUT can be seen in Table 5. It is important to note that, given that the previous approach of Morís et al. [34] only performs image generation using the Montgomery and the Shenzhen datasets, both the results of the baseline and the CUT were obtained for the purposes of this work. Particularly, the results depict an improvement of the performance when adding the generated images, which is reflected in a higher mean accuracy (that goes from 79.33% and 80.19% in the case of the Baseline and the CUT, respectively, to an 82.19% in the case of the Stable Diffusion approach), specificity (going from around 65% in both baseline and CUT to a 72.47% with the Stable Diffusion approach), precision (raising from around 82% in both the baseline and the CUT approach to an 84.50% with the Stable Diffusion approach) and F1-Score, as seen in the performance evolution previously discussed. A notable performance improvement can also be seen in terms of AUC, having a mean value of 0.7646 in the case of the baseline, 0.7683 in the case of CUT but raising to 0.7982 for the Stable Diffusion approach. Finally, in the case of the recall, there is a slight performance drop (from 87.76% with the CUT to 87.17% with Stable Diffusion) that, however, improves the baseline (85.02%). At the same time, it is important to note that the mean performance is very similar and the standard deviation improves (from 13.45% with the CUT to 10.72% with Stable Diffusion).

**Table 5:** Comparison of the results obtained for the automatic lung nodule screening using the JSRT dataset among approaches: the baseline, the data augmentation with the CUT and the data augmentation with the highest-performing configuration of Stable Diffusion (our proposal)

Method	ACC	RECA	SPEC	PREC	F1-SC	AUC
Baseline	79.33% ± 4.05%	85.02% ± 1.77%	65.42% ± 11.70%	82.56% ± 4.91%	83.70% ± 2.90%	0.7646 ± 0.0917
CUT	80.19% ± 4.94%	**87.76%** ± **13.45%**	65.91% ± 17.93%	82.75% ± 7.74%	84.01% ± 5.84%	0.7683 ± 0.0493
Ours	**82.19%** ± **5.96%**	87.17% ± 10.72%	**72.47%** ± **10.96%**	**84.50%** ± **4.10%**	**85.43%** ± **5.86%**	**0.7982** ± **0.0490**

The highest values of each metric (i.e., those with the highest mean among approaches) are highlighted in bold

### Qualitative analysis of the generated images

**Fig. 9:**
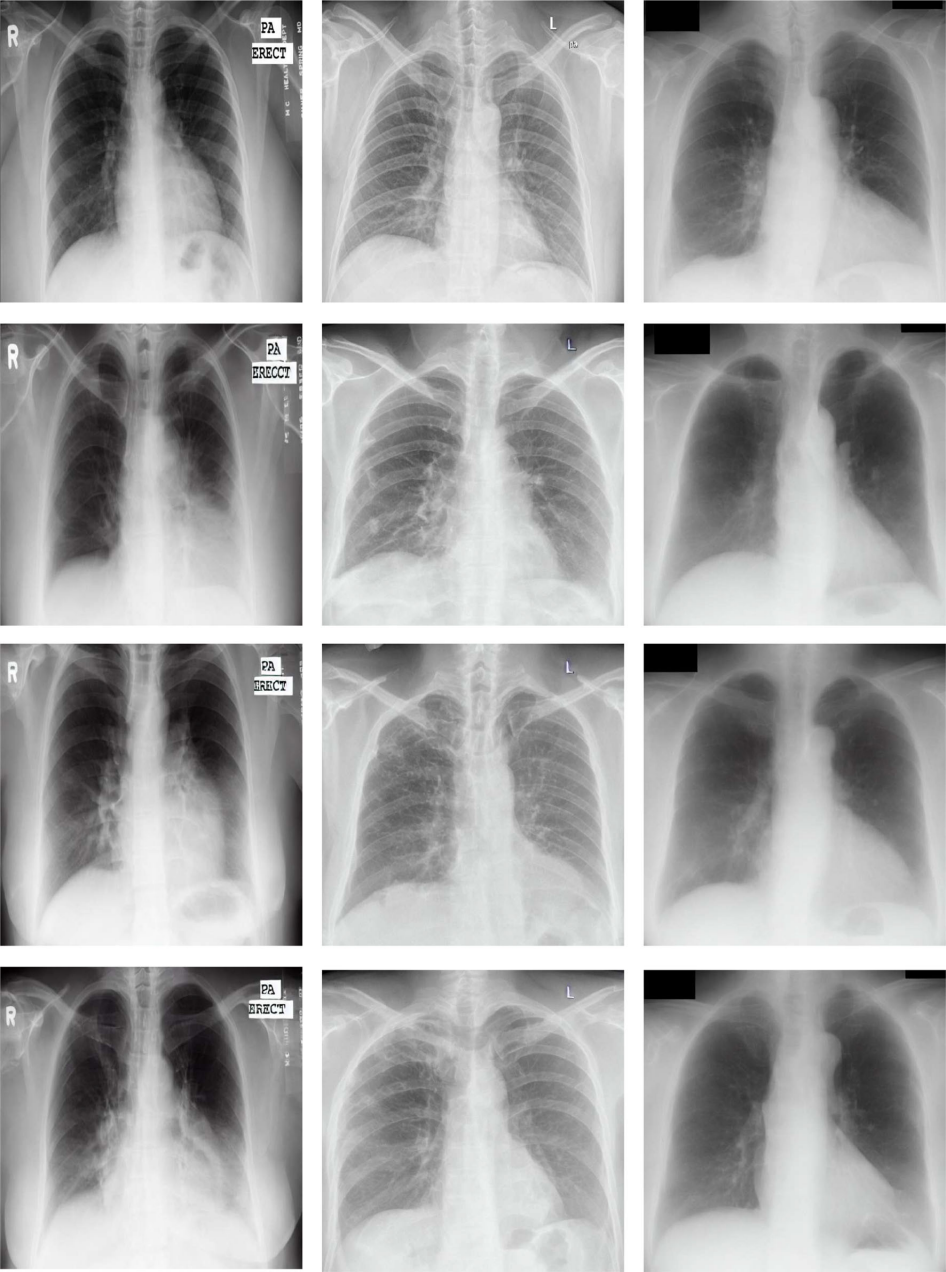
Representative examples of pathological images generated from normal cases by the Stable Diffusion (SD) model. Each column presents the examples of the Montgomery County dataset, Shenzhen and JSRT, respectively. First row: original normal image. Second row: pathological image generated by SD trained 5000 steps. Third row: pathological image generated by SD trained 7500 steps. Fourth row: pathological image generated by SD trained 10,000 steps

Finally, we discuss the quality of the generated images for each of the datasets and scenarios. For each dataset, we compare an original image with some of their corresponding most representative generated images (particularly, trained during 5000, 7500 and 10,000 steps). Moreover, the image quality must also be evaluated in the 2 different generation scenarios: the scenario where normal images are converted to pathological and the scenario where pathological images are converted to normal. The first scenario is evaluated with the images that are provided in Fig. 9. There, it can be seen that the Stable Diffusion model generates images with a realistic appearance in comparison with an original chest X-ray. In addition, the model’s ability to transform various parts of the image in a coherent manner is remarkable, specially in the lung zones, the region of interest in this problem. Another remarkable aspect of the image generation is that the model not only changes the textures of the image but also the shape of the lungs and the diaphragms. Added to this, the model is also able to remove or introduce some structures like those that can be seen below the left lung. This aspect can be associated with the Variational Autoencoder module that composes the Stable Diffusion. This module makes the model able to derive some important features from the whole set of images (such as the previously-mentioned shape of the lungs). Therefore, when the model generates a new image, it randomly selects a coherent value within the normal distribution of the learned features.

**Fig. 10:**
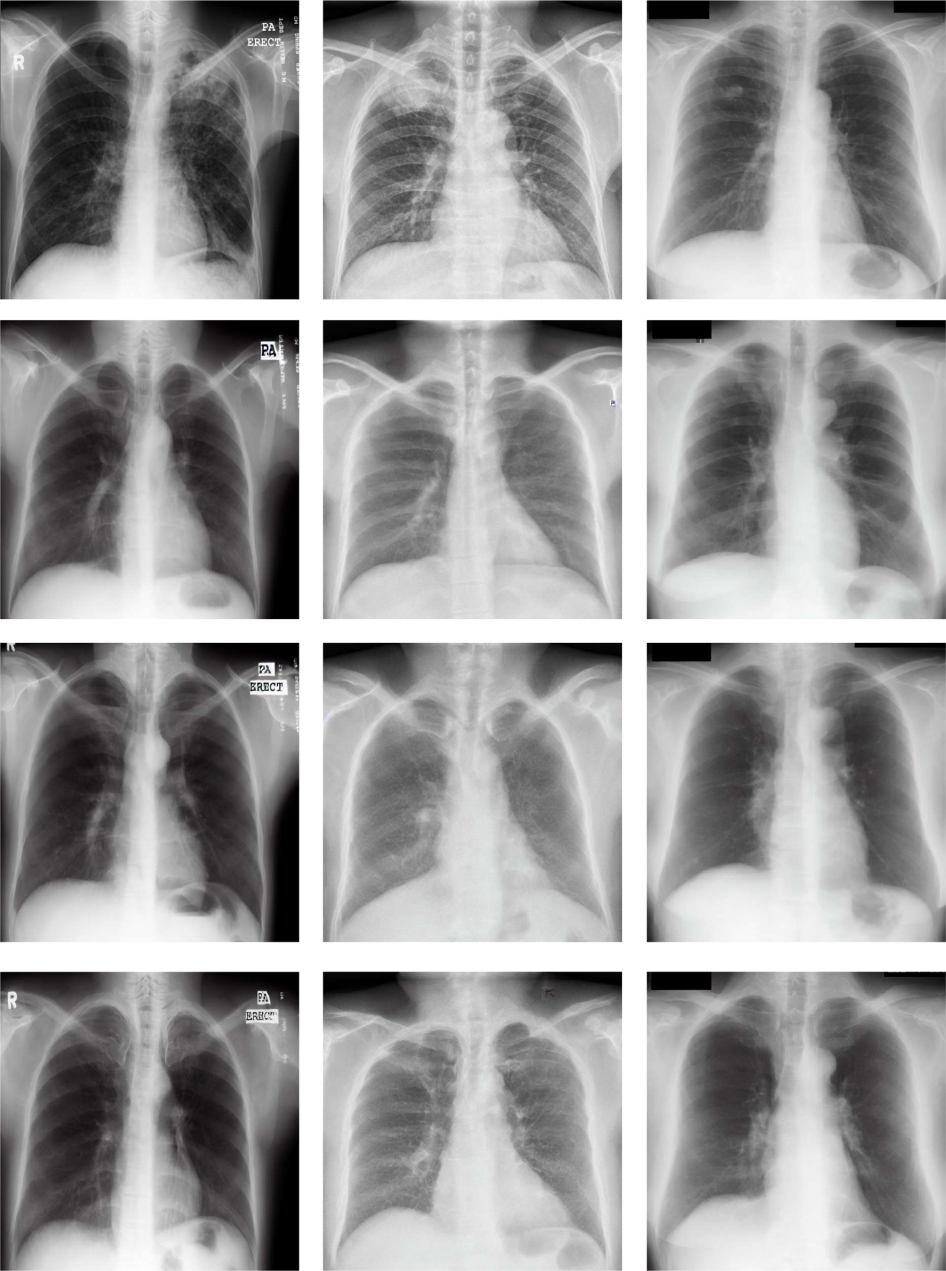
Representative examples of normal images generated from pathological cases by the Stable Diffusion (SD) model. Each column shows the examples of the Montgomery County, Shenzhen and JSRT datasets, respectively. First row: original pathological image. Second row: normal image generated by SD trained 5000 steps. Third row: normal image generated by SD trained 7500 steps. Fourth row: normal image generated by SD trained 10,000 steps

The results of the second scenario can be seen in Fig. 10. In this case, similar discussions can be made. Firstly, the Stable Diffusion model is able to generate images with a realistic appearance, very similar to an original chest X-ray sample. Moreover, the shapes of the lungs have a remarkable variability, an aspect that is once again representative of the normal distribution that has been learned by the Stable Diffusion model regarding several relevant features. Another interesting aspect that can be extracted from the generated images is the capability of the model to remove lung lesions, as expected.

**Fig. 11:**
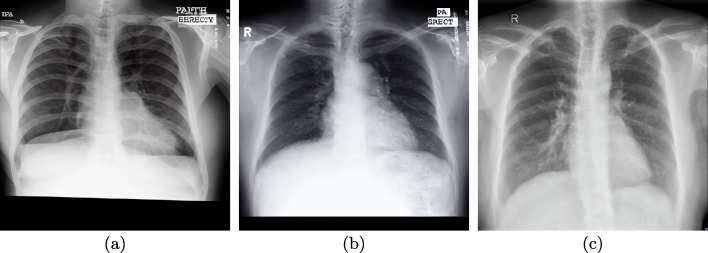
Examples of low quality images generated by Stable Diffusion. (a) Image generated by a Stable Diffusion model that was trained a small amount of training steps. (b) Image generated by a Stable Diffusion model that was trained during a too high number of training steps. (c) Generated image with lack of anatomical coherence, which displays 4 clavicles

Other interesting aspect that can be extracted from the experimentation is that the Stable Diffusion model can assume the global aspect of a chest X-ray with a small amount of images and training steps. Nevertheless, in these situations, the appearance of the generated chest X-ray images exhibits artistic characteristics, as this was the main intention of the original Stable Diffusion proposal. Furthermore, it is also remarkable that too much training steps leads to an important degradation of the image quality, adding a considerable amount of noise and artifacts. This demonstrates the need for the presented ablation study to determine the most appropriate number of training steps. Some examples of images that present these issues can be seen in Fig. 11. It is relevant to mention the fact that this number of training steps depends on the used dataset, as demonstrated in the proposed experimentation. Lack of anatomical coherence is another problem that can be found in some images. This usually affects the shape or the number of some body parts, like the clavicles or the ribs. Despite the associated problematic, this should not affect the performance of the screening models, given that the relevant clinical findings of both analyzed affectations are exclusively found on lung regions. Another issue that can be found on generated images, regardless of the training steps, is the generation of text within the images given that, in many cases, those pieces of text are unreadable. This is a well-known problem with Stable Diffusion but that, however, can be ignored for this particular problem, given that the text labels seen in the chest X-ray images are irrelevant for the automatic screening.

**Table 6:** Comparison in terms of accuracy between our proposal and previous works find in the state-of-the-art for automatic tuberculosis screening

Method	Montgomery County	Shenzhen
Shu et al. [56]	–	84.00%
Ali et al. [57]	–	88.60%
Sirshar et al. [58]	76.08%	76.73%
Hwang et al. [59]	67.40%	83.70%
Zeyu et al. [60]	85.70%	91.20%
Lopes et al. [61]	82.60%	84.70%
Pasa et al. [52]	79.00%	84.40%
Jaeger et al. [62]	78.30%	84.10%
Alfadhli et al. [63]	79.10%	–
Rajaraman et al. [64]	92.30% ± 3.12%	88.79% ± 2.47%
Morís et al. [34]	88.41% ± 5.27%	90.33% ± 1.41%
Ours	97.09% ± 1.46%	92.14% ± 2.22%

### Discussion of the state-of-the art results

Our proposed methodology showcases its robustness and adaptability by leveraging a well-established state-of-the-art architecture for pathological screening, enabling a close comparison with previous reference approaches for data augmentation in the context of chest X-ray imaging. Although our data augmentation methodology based on adapted generative latent diffusion models could be applied to other architectures, it is essential to note that this is beyond the scope of this work. Numerous efforts have been made to develop automatic methodologies for pathological screening using chest X-ray images, employing various pipelines and training configurations, which makes direct comparisons difficult. Furthermore, some works do not include the standard deviation, and/or it is unclear whether they report the mean or the best value of several repetitions. Nevertheless, we provide a thorough comparison of our methodology with previous state-of-the-art strategies for the two pathological scenarios examined in this work, to demonstrate the improvement in terms of performance and the great potential of latent diffusion image generation over other data augmentation methods.

In particular, the proposed adapted generative latent diffusion data augmentation strategy is able to enhance the performance of previous state-of-the-art automatic screening approaches and alternative data augmentation methods. Regarding the case of automatic tuberculosis screening, an interesting discussion can be provided. The comparison of the results is specified in Table 6. There, it can be seen that our approach presents a high performance in comparison with the rest of the contributions of the state-of-the-art. Given that both Montgomery County and Shenzhen datasets provide lung segmentation ground truth, many of the works propose methodologies for such task. Nevertheless, other contributions are also in line with the automatic screening that is being tackled in this work. As reference, the work from Shu et al. [56], which propose an ensemble of features obtained from different deep models and only use the Shenzhen dataset, achieves an accuracy of 84.00% in contrast with the 92.14% obtained in our particular case. Ali et al. [57] propose the use of Incremental Modular Network Synthesis for several medical imaging problems, being tuberculosis screening one of them, using only the Shenzhen dataset. In the comparison, this work obtains 88.60% of accuracy against the 92.14% obtained by our methodology. The work of Sirshar et al. [58] leverages the approach of incremental learning to recognize multiple pulmonary pathologies, being Montgomery County and Shenzhen among the used datasets. In particular, this work obtains a 76.08% of accuracy against the 97.09% of our methodology for the Montgomery County dataset and a 76.73% of accuracy for the Shenzhen dataset compared to our 92.14%.

Hwang et al. [59] propose the use of a deep convolutional neural network for automatic tuberculosis screening, exploiting the transfer learning pipeline, which goes from 67.40% of accuracy to 97.09% for Montgomery County and that goes from 83.70% to 92.14% for Shenzhen. When comparing the performance of our approach with Zeyu et al. [60], a work that contributes proposing a knowledge distillation methodology based on Grad-CAM and enhancing the performance with a prior stage of lung region segmentation, something similar can be obtained, with a performance improvement from 85.70% to 97.09% in the case of Montgomery County and from 91.20% to 92.14% in the case of Shenzhen. Lopes et al. [61] propose several alternatives to leverage features extracted from convolutional neural networks for tuberculosis detection in chest X-ray images. Comparing performances, this contribution obtains 82.60% for Montgomery County (significantly lower than our 97.09%) and 84.70% for Shenzhen (lower than our 92.14%). In the scenario of comparing Pasa et al. [52], which offers the original performance results when using the same screening architecture that we are using in this work, the accuracy raises from 79.00% to 97.09% for the Montgomery County dataset and from 84.40% to 92.14% in the case of Shenzhen. In the case of Jaeger et al. [62], their authors propose the use of classical machine learning approaches to perform an automatic tuberculosis screening extracting texture and shape features from chest X-ray images. In particular, the accuracy is 78.30% for Montgomery County (much lower than our 97.09%) and 84.10% for Shenzhen (notably lower than our 92.14%). Another work we should compare with is Alfadhli et al. [63], where the authors propose the use of Speed-up Robust Features (SURF) descriptors to train a Support Vector Machine. In the same line with the previous works, our proposal obtains a considerably better results in terms of accuracy. In particular, the performance achieved for the Montgomery County dataset (the only dataset that is both used in the mentioned study and in ours as well) is only 79.10% against our 97.09%.

Moreover, Rajaraman et al. [64] propose a methodology of bone suppression on chest X-ray images with the aim to improve the performance of the automatic tuberculosis screening. The accuracy comparison shows an improvement of our proposal, with a raise from 92.30% ± 3.12% to 97.09% ± 1.46% for Montgomery County and from 88.79% ± 2.47% to 92.14% ± 2.22% in the case of Shenzhen. Finally, the proposed data augmentation strategy also improves the performance achieved in [34] with the support of the CUT architecture for image generation, as showcased in the previous sections. When evaluating the methodology with the Montgomery County dataset, this improvement implies a raise from 88.41% ± 5.27% to 97.09% ± 1.46% in terms of accuracy. Moreover, when evaluating with the Shenzhen dataset, the improvement can also be seen in terms of the same global metrics. Specifically, the accuracy goes from 90.33% ± 1.41% to 92.14% ± 2.22%. Globally, the provided comparisons point out the robustness of our methodology and the competitive performance of the data augmentation strategy. Added to this, our pipeline is flexible and easily adaptable to other medical imaging domains, while some presented contributions are strictly limited to the problem they are leading with.

In the case of the lung nodule screening, the analysis obtains that the performance of the model is in line with the results reported by the state-of-the-art in similar tasks. Firstly, it is necessary to remark that the JSRT dataset is generally used for lung segmentation, nodule segmentation or nodule detection rather than screening. Secondly, the works of the state-of-the-art usually slightly modify the original JSRT dataset, while we decided to use it directly in our proposal, without performing additional modifications to its structure. Both aspects make it difficult to find relevant works, but we have made an effort to provide the fairest comparisons possible. As reference, Ausawalaithong et al. [65] propose a methodology for lung nodule detection training a DenseNet-121 model that was pre-trained in ImageNet. Apart from using the JSRT dataset, they also consider a larger dataset known as ChestX-ray14 [66]. The methodology includes 3 different scenarios. In the first scenario, the model is trained and tested with the ChestX-ray14 dataset while, in the second scenario, the process is done training and testing with the JSRT dataset. Finally, in the third scenario, the model is sequentially trained on ChestX-ray14, then on JSRT and finally it is tested with the JSRT dataset. While comparing this approach with our proposal, we only consider the second scenario, as it fits with our particular case (training and testing only with JSRT dataset). In particular, the accuracy goes from 65.51% to 82.19%. Nonetheless, it is important to point out that the aim of this state-of-the-art contribution is to recognize lung cancer while our reported results are obtained from distinguishing between control cases and cases with lung nodules, which can be either malignant or benign.

On the other hand, the work of Li et al. [67], which uses 2 slightly modified versions of the JSRT dataset and a complex pipeline of image enhancement and preprocessing, obtains a recall of 90% for the first version of the dataset (named as JSRT A) and of 93% for the second one (named as JSRT B). In this context, our proposed methodology obtains a competitive recall of 87.17%. We can also include the work of Li et al. [68], which uses the same 2 modified versions of the JSRT dataset (JSRT A and JSRT B). The authors demonstrate a high performance, with an AUC of 0.982 and 0.987, respectively. Nevertheless, this performance is achieved with a complex and ad hoc pipeline with lung field segmentation and rib suppression with active shape models, image enhancement, nodule detection with postprocessing and multi-resolution patch extraction. Finally, these extracted patches are fed to an appropriate convolutional network architecture. This tailored pipeline has a restricted flexibility, making it difficult to extrapolate the methodology to other problems, specially due to the specific image preprocessing steps carried out. In contrast, our work presents a flexible methodology that can be easily adapted to other medical imaging domains. This adaptability allows for the integration of data augmentation strategies into any pipeline and proposal, ultimately providing a more versatile and efficient solution for a wider range of applications.

## Conclusions

In this work, we have proposed a data augmentation strategy based on a latent diffusion model, the popular Stable Diffusion, for chest X-ray image generation, aiming to improve automatic screening for two different respiratory disease pathological scenarios with global impact (tuberculosis and lung nodules). We utilized three datasets representative of data scarcity scenarios, two public datasets for tuberculosis screening (Montgomery County and Shenzhen), and one for lung nodule screening (JSRT). We conducted three exhaustive experiments, one for each dataset, with four different configurations for the generation model (trained after 2,500, 5,000, 7,500, and 10,000 steps, respectively) and an additional configuration of 12,500 steps for the JSRT dataset to ensure the convergence of global performance. The results were compared using an incremental approach, studying the performance of the baseline (i.e., with classical data augmentation), CUT augmentation, and the latent diffusion-based augmentation. Our results demonstrate that the latent diffusion-based approach can generate useful images representative of the scenarios to be modeled (both normal and pathological) while applying coherent transformations on relevant image regions, such as the lungs. The model’s ability to learn relevant features like the lung shape and reflect them in the novel set of generated images is also shown. Overall, the use of the latent diffusion-based data augmentation strategy resulted in improved accuracy across all three datasets.

As future work, the image generation process could be performed by training a single model with different plain prompts or even prompts provided by specific medical reports, following state-of-the-art trends that exploit natural language in image analysis scenarios. This approach would leverage the text encoder module and the advantages offered by image generation architectures incorporating natural language processing. Another alternative could be to replace the original text encoder with a more domain-specific one (e.g., trained on biomedical text datasets), guiding the training and inference processes more precisely. Furthermore, exploring other training frameworks different from DreamBooth could be valuable, such as unfreezing the Variational Autoencoder to allow learning specific characteristics of chest X-ray images. The refinement of this component could be helpful to improve the quality of the generated images globally, and specifically to address the issue of anatomical incoherence that can be seen in some images. Another line of interest in future work could be to investigate the performance impact of this image generation strategy with other network architectures for automatic screening. Lastly, it is worth noting that the results obtained in this work could be extrapolated to other medical imaging modalities and domains.

## Data Availability

The associated datasets of the current study are publicly available.
